# Antcin K ameliorates cardiotoxin-induced skeletal muscle injury and inflammation via IL-10 regulation

**DOI:** 10.7150/ijbs.107343

**Published:** 2025-03-19

**Authors:** Ting-Kuo Chang, Lin-Chu Huang, Yueh-Hsiung Kuo, Chun-Hao Tsai, Hsien-Te Chen, Yi-Syuan Wu, Chih-Hsin Tang, Chen-Ming Su

**Affiliations:** 1 Department of Medicine, Mackay Medical College, New Taipei, Taiwan.; 2 Division of Spine Surgery, Department of Orthopedic Surgery, MacKay Memorial Hospital, New Taipei, Taiwan.; 3 Department of Sports Medicine, China Medical University, Taichung, Taiwan.; 4 Department of Chinese Pharmaceutical Sciences and Chinese Medicine Resources, China Medical University, Taichung, Taiwan.; 5 Chinese Medicine Research Center, China Medical University, Taichung, Taiwan.; 6 Department of Orthopedic Surgery, China Medical University Hospital, Taichung, Taiwan.; 7 School of Medicine, China Medical University, Taichung, Taiwan.; 8 Spine Center, China Medical University Hospital, China Medical University, Taichung, Taiwan.; 9 Department of Pharmacology, School of Medicine, China Medical University, Taichung, Taiwan.; 10 Department of Medical Laboratory Science and Biotechnology, College of Medical and Health Science, Asia University, Taichung, Taiwan.

**Keywords:** Antcin K, muscle injury, IL-10, cardiotoxin, inflammation

## Abstract

**Background:** Skeletal muscle, functioning as an endocrine organ, produces a variety of molecules that contribute to the pathophysiology of sarcopenia, leading to muscular injury and inflammation. Antcin K, a bioactive compound derived from *Antrodia cinnamomea* and used in traditional Chinese medicine for its anti-inflammatory properties, was evaluated in this study with the aim of assessing its effects on resisting the progression of sarcopenia both *in vitro* and *in vivo*.

**Methods:** Cardiotoxin (CTX)-induced muscle injury and the treatment of Antcin K in C2C12 cells were both used for RNA sequencing and ingenuity pathway analysis. We also stably cloned an IL-10 knockdown (IL-10^-/+^) C2C12 cell line for the effects of Antcin K treatment on CTX-induced muscle injury. CTX-induced muscle injury in a mouse model.

**Results:** Antcin K ameliorated the CTX-induced muscle injury and inflammation in myoblasts and differentiated myocytes. Bioinformatics analysis results demonstrated the ability of Antcin K to modulate inflammation and enhance myogenesis via upregulated IL-10. Antcin K enhances IL-10 production via the PI3K/Akt signaling pathways. For the *in vivo* results, Antcin K protects against CTX-induced skeletal muscle inflammation and injury.

**Conclusion:** Antcin K ameliorated CTX-induced muscle injury and inflammation through PI3K and Akt and upregulated IL-10 *in vitro*. The CTX-induced injury mouse model was rescued by intraperitoneal injection of Antcin k *in vivo*. Antcin K shows promise as a prospective candidate for the development of an innovative treatment for muscular injury, with significant implications for sarcopenia.

## Introduction

Skeletal muscle, functioning as an endocrine organ, produces a variety of molecules that contribute to the pathophysiology of sarcopenia 1. Sarcopenia, characterized by skeletal muscle atrophy and functional impairment, results from a pathological muscle injury and imbalance between muscle protein synthesis and degradation rates or inflammation 2-4. Muscle injury-induced atrophy is marked by the expression of atrophic markers like muscle RING-finger-1 (MuRF1) and Atrogin-1 5, 6, while skeletal muscle features key myogenic regulatory factors like myogenic differentiation (MyoD) and paired box 7 (Pax7), which are essential for promoting myogenesis and muscle differentiation 7. During myogenesis, myoblasts differentiate into multinucleated myocytes, which express various conserved cytoplasmic proteins, including myosin heavy-chain (MyHC) and desmin 8, 9. Therefore, it is critical to find a therapeutic target or develop treatment strategies for muscle injury or sarcopenia.

Inflammation is a significant factor in the pathogenesis of muscle injury, strongly implicated in its development and closely linked to musculoskeletal damage 10, 11. Pro-inflammatory cytokines are key contributors to chronic inflammation found in inflammatory myopathies that display skeletal muscle injury 12 and are involved in the process of pathological pain 10. Interleukin (IL)-6 and IL-1β have been shown to promote muscle injury via blunting muscle anabolism and energy homeostasis 13. In addition, previous study has shown that immune response is recruited by the cardiotoxin (CTX)-induced muscle injury 14. Inflammatory myopathy symptoms are observed in the environment of CTX-induced murine myoblasts 15. Anti-inflammatory cytokines, such as IL-10 and IL-4, play an important role in the pathophysiology of muscle injury 16. IL-10 is a well-known cytokine for inhibiting IL-6 and IL-1β and is thought to alleviate clinical inflammation symptoms via hematopoietic cells 17-19. A recent study has demonstrated that IL-10 knockout mice had a lower muscle strength after acute exercise 20. However, the relationship between skeletal muscle injury and inflammation is still unclear, highlighting the importance of identifying therapeutic targets and developing treatment strategies for muscle injury and sarcopenia.

Several studies have revealed the plant-derived natural compound that regulates the effects of sarcopenia 21. Antcin K, derived from *Antrodia cinnamomea* (*A. cinnamomea*), has a long history in traditional Chinese medicine for disease and has been shown to possess hepatoprotective properties by modulating oxidative stress 22-25. Antcin K has been shown to have a wide range of pharmacological actions in experimental tests, such as immunomodulatory, anti-inflammatory, and anti-cancer 24. Studies indicate that Antcin K protects against the release of IL-1β, IL-6, and TNF-α in human rheumatoid synovial fibroblasts (RASFs) 26. Hence, it is crucial to discover methods for mitigating sarcopenia by suppressing inflammation. Nevertheless, the role of Antcin K in muscle injury or sarcopenia remains unexplained. In this study, we initially investigated the underlying mechanisms by which Antcin K exerts its effects on CTX-induced muscle injury and inflammation in murine myoblasts and differentiated myocytes *in vitro*. Finally, we employed a CTX-induced mouse model for muscle injury and inflammation to explore the role of Antcin K *in vivo* and assess whether above mechanism could be translated into a potential therapeutic strategy.

## Materials and methods

### Materials

All the antibodies and primers are shown in the Supplementary File.

### Cell culture and stable knockdown cell clones

Both myoblast cell lines, C2C12 and G7, were purchased from the American Type Culture Collection (Manassas, VA, USA) and were incubated in 5% CO_2_ at 37 °C. Both myoblast cell lines were cultured in Dulbecco's Modified Eagle's Medium (DMEM) (Gibco, Grand Island, NY, USA) supplemented with 10% fetal bovine serum (Gibco, USA) containing antibiotics (100U/ml penicillin, 100µg/mL streptomycin). Both myoblast cell lines were cultured in the differentiated medium (DM) which is DMEM with 2% horse serum medium for 3~5 days to differentiate into myocytes, and the DM was changed every day before the following Antcin K treatment or CTX stimulation. The IL-10-/+ C2C12 cell line was stably cloned using a lentivirus, according to the previous study 27.

### Bioinformatics analysis

Based on data from the Gene Expression Omnibus (GEO) dataset, the GSE1428 dataset analyzed the vastus lateralis muscle in young (aged 19-25 years) and older (aged 70-80 years) male subjects. RNA sequencing uses the capabilities of high-throughput sequencing methods to provide insight into the transcriptome of a cell. Ingenuity Pathway Analysis (IPA) was used to predict the activation or inhibition state of the canonical pathway, and it was analyzed based on the reported literature.

### CTX-induced muscle injury mouse model

Eight-week-old male C57BL/6 mice were purchased from the National Laboratory Animal Centre (Taipei, Taiwan). The protocol of animal use has been reviewed and approved by the Institutional Animal Care and Use Committee at China Medical University (the certified numbers: **CMUIACUC-2023-148**). CTX has been used to induce skeletal muscle injury or injury in many mouse models 15, 28. The mice were randomly separated into four groups: control group, CTX-induced muscle injury group, CTX-induced muscle injury with Antcin K low dose group (10 mg/kg), and CTX-induced muscle injury with Antcin K high dose group (30 mg/kg) (n=10 for each group). Briefly, CTX (50µL/10µM) was intramuscularly injected into the tibialis anterior (TA) muscle to induce short-term skeletal muscle injury at the Day 0, followed by another intraperitoneal injection of Antcin K low or high dose every two days. The mice were sacrificed and analyzed on Day 6. During the *in vivo* experiments, locomotor function in mice was assessed every two days, including measurements of grip strength, performance in the rotarod test, and body weight.

### Locomotor measurements

To measure the locomotor function for the mice with CTX-induced muscle injury, a rotarod device (Singa Technology Corporation, Taiwan) and the digital grip strength meter (Bio-Cando Incorporation, Taiwan) were used, according to our previous protocols 29. The digital grip strength meter was employed to measure the hind limb grip strength of the mice. For the rotarod assessment, the device was set to a speed of 25 rpm and operated for 30 minutes while the mice ran. These two tests were measured every 2 days before the mice were sacrificed at the Day 6.

### Skeletal muscle tissues analysis

For immunohistochemical staining, TA muscles from hind limbs of the mouse model were prepared for paraffin-embedded sections, according to previous reports 30. The prepared sections were treated with the primary rabbit anti-dystrophin polyclonal antibody (Abcam, Cambridge, UK) and the secondary antibody Alexa-Fluor® 594 conjugate (Thermo Fisher Scientific, Hemel Hempstead, UK) for myofibers stains, and the dystrophin-positive stained myofibers were examined using TissueFAXS® Spectra systems (Tissue-Gnostics, Vienna, Austria) 31. In addition, TA muscles were pre-stained with phosphotungstic acid for nearly 30~45 days, followed by micro-computed tomography (micro-CT) analysis 32.

### Statistical analysis

All the graphical results and statistical evaluation, were conducted with Graph Pad Prism software version 8 (GraphPad Prism Software Inc., San Diego, CA, USA). Data in all figures are presented as the mean ± standard deviation (SD). Differences between selected pairs from the experimental groups were analyzed for statistical significances using the Student's *t*-test. Statistical comparisons among three or more groups or the independent samples used two-way ANOVA. Between-group differences were considered significant if p-values were less than 0.05.

## Results

### Antcin K inhibits CTX-induced inflammation and injury

**Figure 1:**
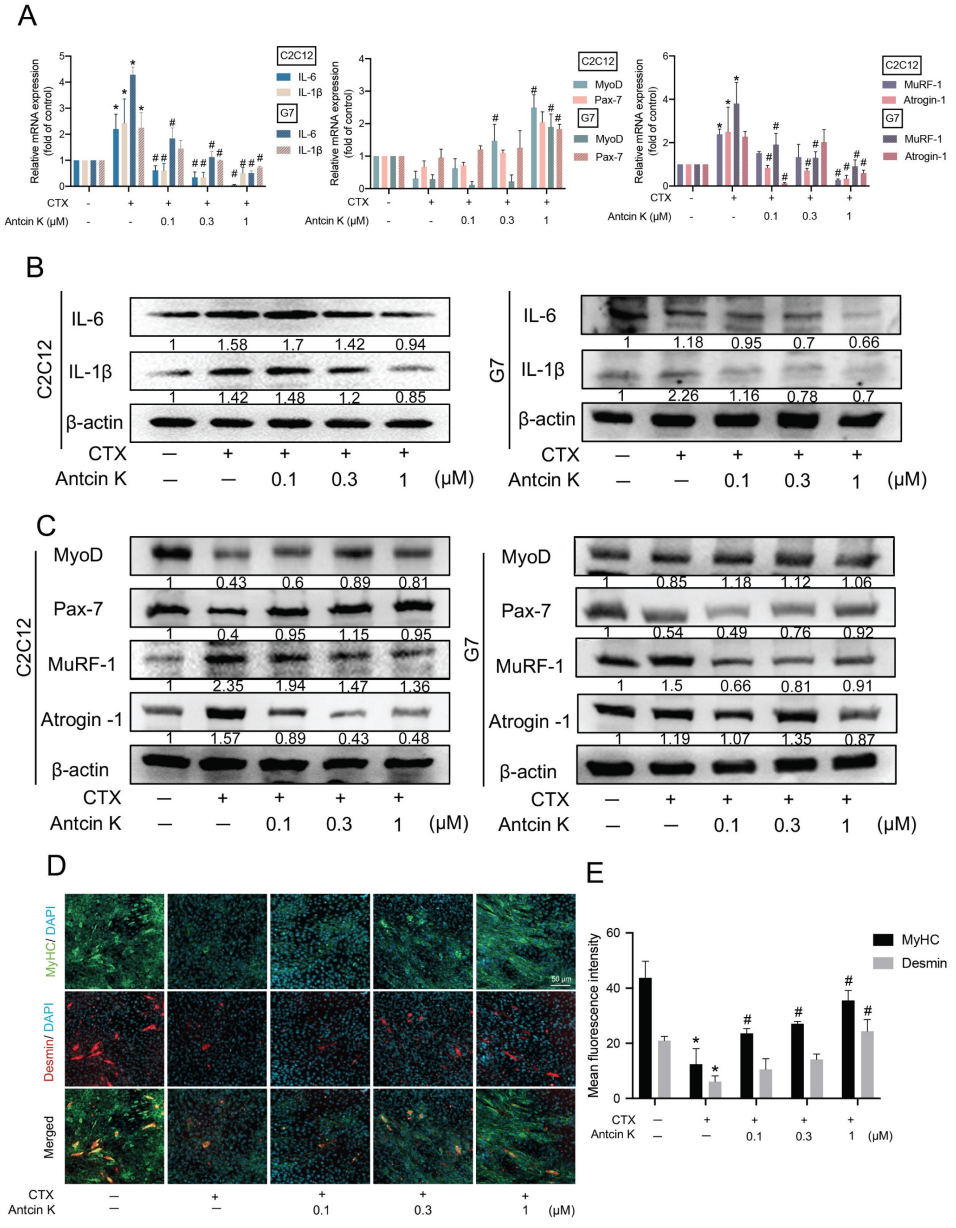
** Antcin K alleviated CTX-induced inflammation and enhanced differentiation. (A, B, C)** The C2C12 and G7 were treated with the CTX (0.5 µM) for 24 h, followed by Antcin K (0, 0.1, 0.3, and 1 µM) for another 24h. (n=3). The mRNA and protein expression of proinflammatory cytokines (IL-6, IL-1β), myogenesis (MyoD, Pax-7), and atrophy markers (MuRF-1, Atrogin-1) were measured by qRT-PCR **(A)** and western blotting **(B, C)** on C2C12 and G7 cell lines. C2C12 myoblasts were cultured to 80% confluent, then were treated with differentiation medium for 3 days, and on the third day, we treated CTX for 24h, followed by treating Antcin K (0, 0.1, 0.3, and 1 µM) to examine the morphology of MyHC and Desmin **(D)**. Quantified analysis by Image J software (version 1.53) confirmed the mean fluorescence intensity of **(E)** MyHC and Desmin positive staining. *p < 0.05 compared with the control group, #p < 0.05 compared with the CTX-induced muscle injury group.

CTX is a suitable injury model for skeletal muscles which have relatively low harmfulness for myoblasts of hind limbs 33. To examine the role of Antcin K in CTX-induced muscle injuries, myoblast cell lines were initially used for the mechanical and functional analyses. As shown in Figure 1A, C2C12 and G7 cell lines were separately stimulated with CTX for 24 hours to induce muscle injuries, followed by treatment with Antcin K for an additional 24 hours in a concentration-dependent manner. After inducing muscle injuries with CTX, treatment with Antcin K led to a decrease in pro-inflammatory markers IL-6 and IL-1β, and atrophic markers MuRF-1 and Atrogin-1, as well as an increase in myogenic markers MyoD and Pax-7 **(Figure 1A)**. Also, the treatment with Antcin K also produced similar effects on protein expression in CTX-induced muscle injury **(Figure 1B-C)**. To further examine the impact of Antcin K on differentiated myocytes, C2C12 cells were treated with DM for 4~5 days. Immunofluorescence staining demonstrated that Antcin K rescues the expression of MyHC and Desmin after CTX-induced muscle injury **(Figure 1D)**, and the fluorescent intensity of MyHC and Desmin was separately quantified by ImageJ software **(Figure 1E)**. The above results demonstrated that Antcin K inhibited CTX-induced muscle injury in myoblasts and myocytes.

### Bioinformatics results indicate a regulatory role of IL-10 in the effects of Antcin K treatment on CTX-induced muscle injury

**Figure 2:**
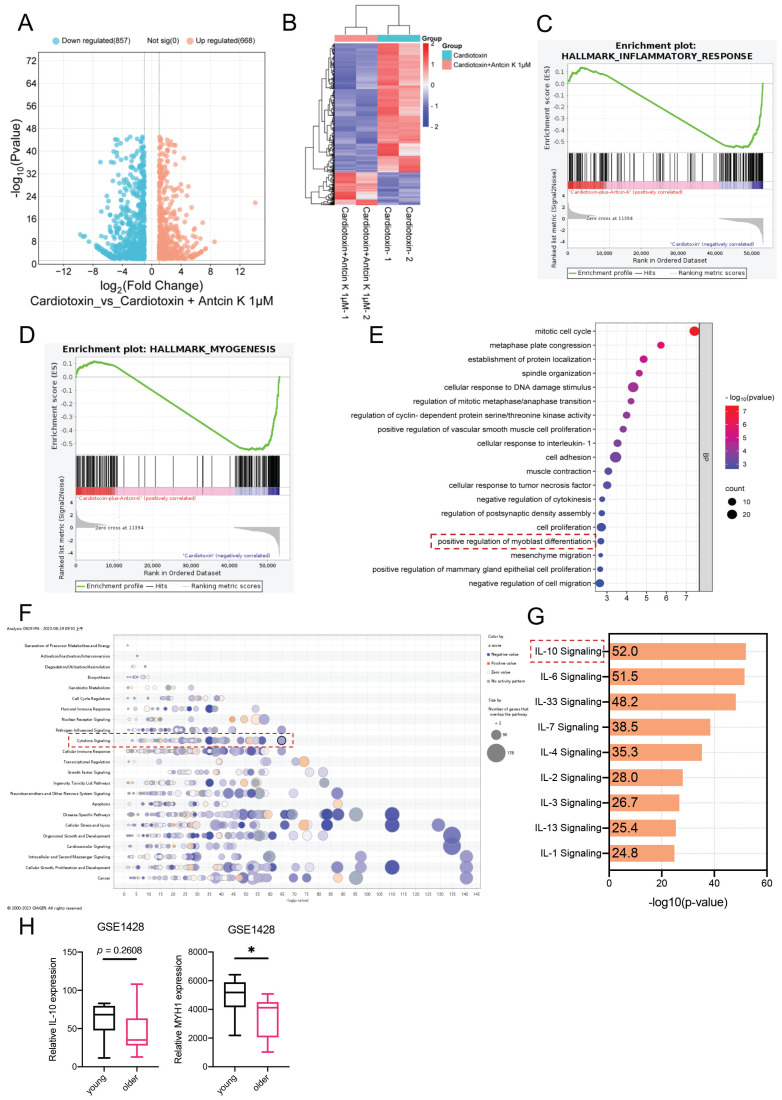
** Bioinformatic results demonstrated that IL-10 is involved in the effects of Antcin K in treating CTX-induced muscle injury. (A)** The volcano plot analysis revealed that the treatment with the Antcin K group demonstrated differentially expressed genes in the CTX-induced injury treatment with the Antcin K group. **(B)** The result of a heatmap of RNA sequencing showing differentially expressed genes in C2C12 cells with or without Antcin K treatment. **(C, D)** The results of the Gene set enrichment analysis (GSEA) plot presented that Antcin K treatment relates to the inflammatory response and myogenesis. **(E)** Kyoto Encyclopedia of Genes and Genomes (KEGG) pathways indicates the enrichment included in myoblast differentiation.** (F)** Ingenuity Pathway Analysis (IPA) evidenced that IL-10 signaling is involved in cytokine signaling in IPA results. **(G)** IL-10 signaling is the highest score in anti-inflammatory cytokine signaling. **(H)** IL-10 and MYH1 expression were expressed in the young and older groups based on the GEO database (GSE1428). *p < 0.05 compared with the young population.

RNA sequencing was used to determine the differential genes in the previous study 29. To further advance exploring the relationship between Antcin K and CTX-induced muscle injury, the C2C12 cells were used for RNA sequencing. In the comparison of CTX group and CTX treating with Antcin K group, the volcano plot demonstrated that 880 genes were significantly expressed **(Figure 2A).** The heat map results have shown that 179 genes were upregulated and 701 genes were downregulated in the CTX treating with Antcin K group **(Figure 2B)**. A Gene Set Enrichment Analysis (GSEA) plot demonstrated a correlation between the myogenesis and inflammatory response between CTX group and CTX treating with Antcin K group **(Figure 2C and D)**. According to the results of RNA sequencing, those differentially expressed genes (DEGs) were subjected to Gene Ontology enrichment analysis and Kyoto Encyclopedia of Genes and Genomes pathway analysis, and the positive regulation of myoblast differentiation was involved in biological processes **(Figure 2E)**. Next, those DEGs were input into the IPA databases and found that the IL-10 signal was the most significant signals in the cytokine signaling **(Figure 2F and G)**. We further investigated the Gene Expression Omnibus (GEO) database to search the expression of IL-10 and various muscle differentiation markers in human populations. The GEO results indicated that the expression of IL-10 had a little different in the young and old human groups, but the expression of MYH1 was significantly decreased in the older human group **(Figure 2H)**. Our results demonstrated that IL-10 was a key regulator for the effects of Antcin K treatment on CTX-induced muscle injury.

### Antcin K facilitated CTX-induced inflammation and injury signals through IL-10 expression

**Figure 3:**
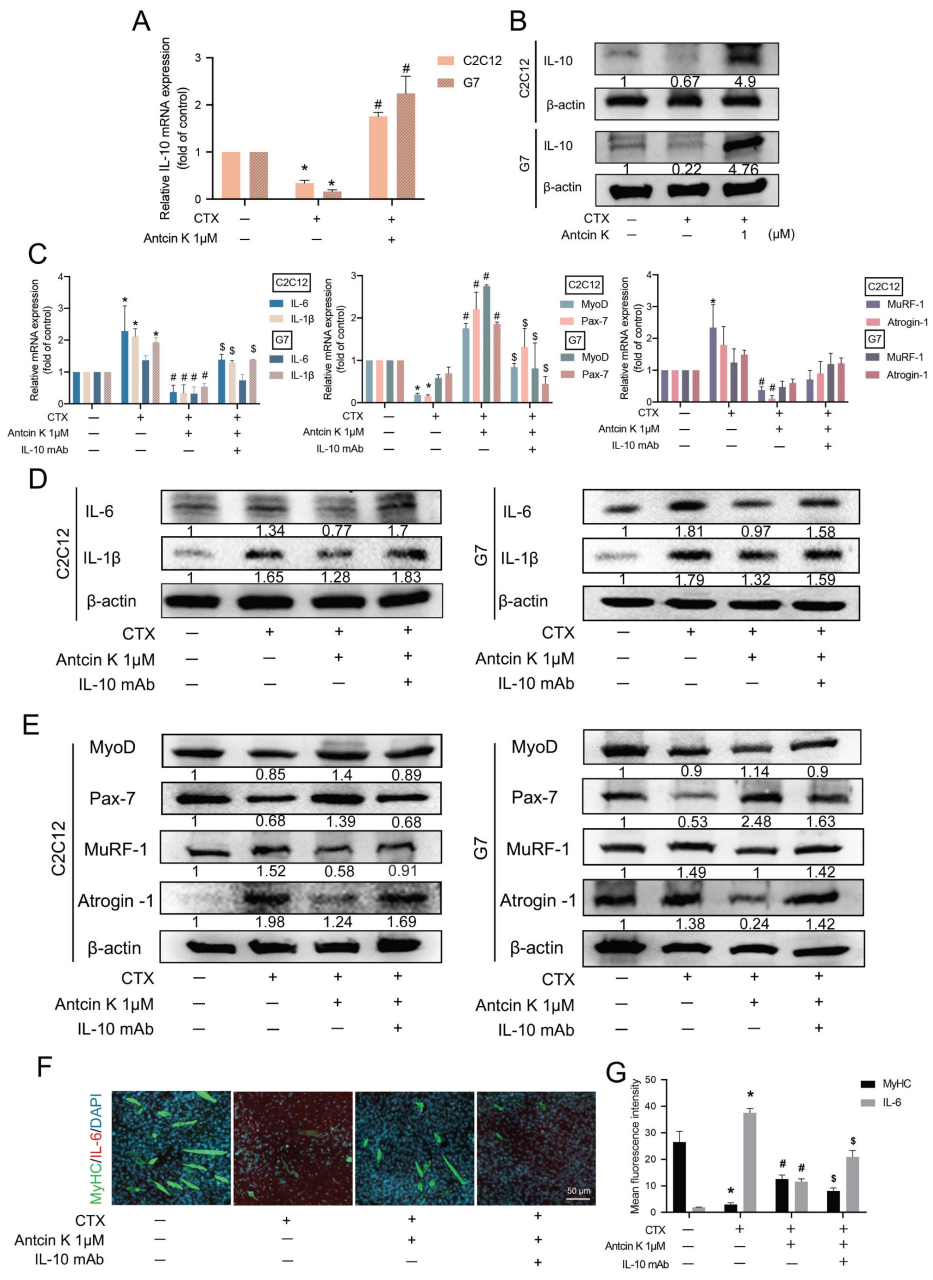
** Antcin K increased IL-10 production and reduced the expression of pro-inflammatory cytokines in murine myoblasts. (A, B)** The C2C12 and G7 cell lines were incubated by the CTX for 24 h and followed treatment with the Antcin K (1µM). **(A)** The level of mRNA of IL-10 was examined by the qRT-PCR. **(B)** Protein expression in the C2C12 and G7 cell lines was checked by western blotting. **(C, D, E)** C2C12 and G7 cell lines were treated with CTX for 24 h, followed by a 30-minute exposure to an anti-IL-10 monoclonal antibody, and Antcin K (1µM) was treated for another 24 h. The mRNA levels **(C)** and protein expression **(D and E)** of proinflammatory markers, IL-6 and IL-1β, myogenesis markers, MyoD, Pax-7, and atrophy markers, MuRF-1, Atrogin-1, were analyzed in C2C12 and G7 cell lines, respectively. **(F)** Immunofluorescence staining demonstrated the expression of skeletal muscle markers MyHC and pro-inflammatory cytokine IL-6. Quantified analysis by Image J software (version 1.53) confirmed the mean fluorescence intensity of **(G)** MyHC and IL-6-positive staining. *p < 0.05 compared with the control group, #p < 0.05 compared with the CTX-induced muscle injury group. $p < 0.05 compared with the CTX + Antcin K treatment group.

A previous study discovered that IL-10 inhibits the production of cytokines such as IL-6 and IL-1β in skeletal muscle 19. To examine the role of IL-10 involved in the treatment of Antcin K after CTX-induced muscle injury, we first examined IL-10 levels in C2C12 and G7 cells for the effects of Antcin K treatment after CTX-induced muscle injury **(Figure 3A-B)**. Next, we used neutralizing anti-IL-10 antibody to further examine the involvement of IL-10 in the treatment of Antcin K after CTX-induced muscle injury. The inhibition of IL-6, IL-1β, MuRF-1, and Atrogin-1 expression, as well as the induction of MyoD and Pax7 expression by Antcin K treatment, were reversed by IL-10 neutralizing antibody in both C2C12 and G7 cell lines **(Figure 3C─E)**. However, the expression patterns of MyoD, Pax7, MuRF-1 and Atrogin-1 are not significant affected in the CTX-induced muscle injury group compared to the control group in G7 cells **(Figure 3C)**. Myoblasts were incubated with DM for 5 days to become myocytes. Immunofluorescence staining demonstrated the expression of MyHC and IL-6 was also reversed by IL-10 neutralizing antibody in C2C12 cells **(Figure 3F)**, and the fluorescent intensity of MyHC and IL-6 was separately quantified by ImageJ software **(Figure 3G)**. These results indicate that Antcin K upregulates IL-10-dependent anti-inflammatory responses and promotes muscle-related markers under CTX treatment.

### Antcin K alleviated CTX-induced muscle injury in endogenous IL-10-/+ cell lines

**Figure 4:**
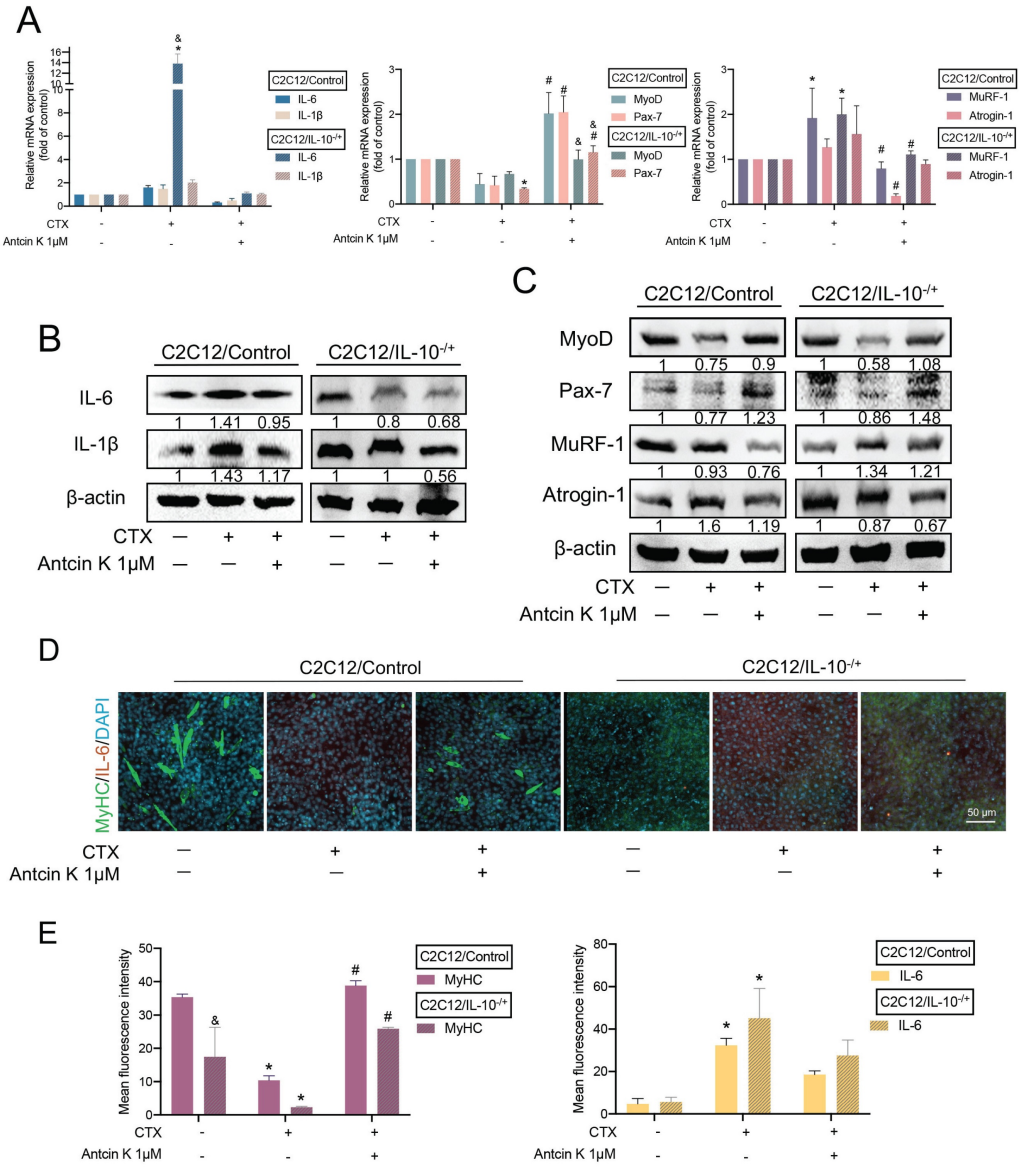
** Antcin K reduced CTX-induced muscle injury and inflammation in IL-10^-/+^ cell lines. (A)** After incubating with CTX (0.5 µM) for 24 h, the wild-type and IL-10^-/+^ were afterward treated with Antcin K (1 µM) for another day. **(B and C)** Protein levels of inflammatory markers (IL-6, IL-1β), myogenesis markers (MyoD, Pax-7), and atrophy markers (MuRF-1, Atrogin-1) were detected by Western blotting.** (D)** Immunofluorescence staining showed the expression of skeletal muscle markers MyHC and the pro-inflammatory cytokine IL-6. **(E)** The mean fluorescence intensity of MyHC and IL-6 was quantified by using Image J software (version 1.53). *p < 0.05 compared with the control group, #p < 0.05 compared with the CTX-induced muscle injury group. &p < 0.05 indicates a significant difference between C2C12/IL-10^-/+^ and C2C12/Control cell lines within the same experimental group.

Additionally, we further knocked down IL-10 expression endogenously in C2C12 cells so we stably cloned an IL-10 knockdown C2C12 cell line (IL-10^-/+^), and confirmed its efficiency by using quantitative real-time polymerase chain reaction and western blots **(Supplementary Figure S1)**. Next, we incubated with CTX for 24 hours, followed by treatment with Antcin K for another 24 hours both in C2C12/Control and C2C12/IL-10^-/+^ cells. After inducing muscle injuries with CTX, treatment with Antcin K led to a decrease in IL-6, IL-1β, MuRF-1, and Atrogin-1, as well as an increase in MyoD and Pax-7 in the C2C12/IL-10^-/+^ cells compared to C2C12/Control cells **(Figure 4A)**. Also, the result of those protein expression had the similar effects in the C2C12/IL-10^-/+^ cells compared to C2C12/Control cells **(Figure 4B and C).** To further examine the endogenous role of IL-10 involving in the impact of Antcin K on differentiated myocytes, C2C12/Control and C2C12/IL-10^-/+^ cells were both treated with DM for 5 days. Immunofluorescence staining demonstrated that Antcin K treatment reversed the increasing MyHC and decreasing IL-6 in C2C12/IL-10^-/+^ cells** (Figure 4D)**, and the fluorescent intensity of MyHC and IL-6 was separately quantified by ImageJ software **(Figure 4E)**. The present results showed that both exogenous or endogenous levels of IL-10 are a key regulator for the effects of Antcin K treatment on CTX-induced muscle injury.

### Antcin K promotes IL-10 production via the PI3K/Akt pathways in CTX-induced muscle injury

**Figure 5:**
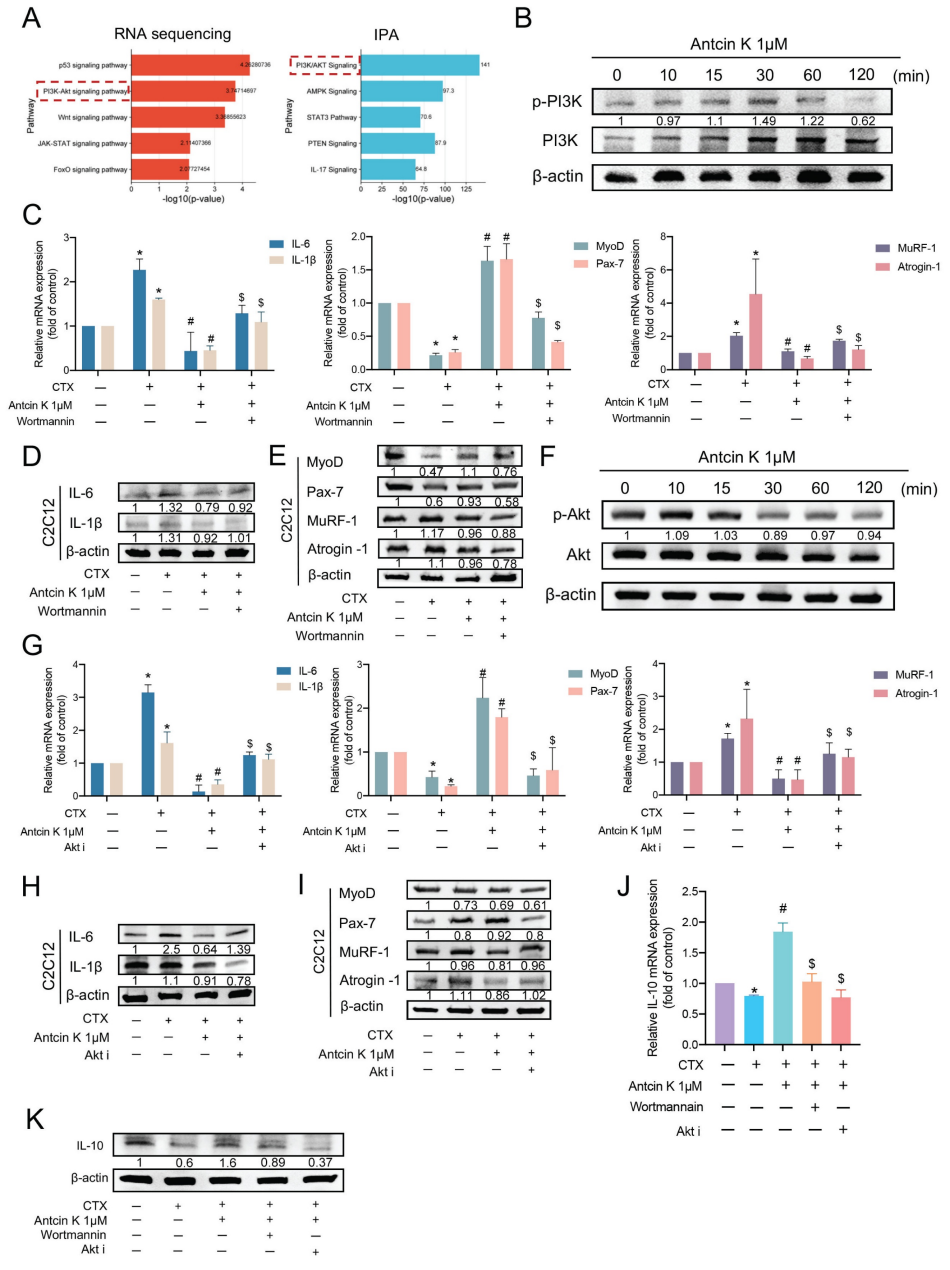
** Antcin K activated the PI3K and Akt pathways in C2C12 through increasing IL-10 production. (A)** The results of RNA sequencing and IPA demonstrated the PI3K/Akt signaling pathway is involved in the CTX-induced injury treatment with the Antcin K group. **(B)** C2C12 cells were incubated with Antcin K (1µM) for the indicated time points, and cell lysates were collected to evaluate the phosphorylation of PI3K levels. **(C, D, E)** Myoblasts were incubated with the CTX for 24 h, then treated with the wortmannin (1nM) for 30 min, followed by Antcin K treatment before, evaluating the mRNA** (C)** and protein levels **(D, E)**. **(F)** C2C12 cells were treated with Antcin K (1µM) for the indicated time points. Cell samples have been taken to evaluate the phosphorylation of AKT levels. **(G, H, I)** C2C12 cells were incubated with CTX for a full day before being treated with an Akt inhibitor for 30 minutes, followed by Antcin K treatment to assess mRNA **(G)** and protein levels **(H, I)**. **(J, K)** C2C12 cells were treated with CTX for 24 h and afterward incubated the wortmannin (10nM), and Akt inhibitor (25nM) for 30 min before treating the Antcin K (1µM). Expression of IL-10 mRNA and protein levels were quantified by **(J)** qRT-PCR and** (K)** western blotting. The Values are mean ± SD of at least three independent experiments. *p < 0.05 compared with the control group, #p < 0.05 compared with the CTX-induced muscle injury group. $p < 0.05 compared with the CTX + Antcin K treatment group.

According to RNA sequencing and IPA results, we aimed to select and elucidate the mechanism underlying the PI3K/Akt signaling pathway for the effects of Antcin K treatment on CTX-induced muscle injury **(Figure 5A)**. Stimulating C2C12 with Antcin K promoted PI3K phosphorylation in C2C12 cells **(Figure 5B)**. Next, we used PI3K inhibitor, wortmannin, to further examine the involvement of PI3K in the treatment of Antcin K after CTX-induced muscle injury. The inhibition of IL-6, IL-1β, MuRF-1, and Atrogin-1 expression, as well as the induction of MyoD and Pax7 expression by Antcin K treatment, were reversed by PI3K inhibitor in C2C12 cells **(Figure 5C─E)**. Stimulating C2C12 with Antcin K promoted Akt phosphorylation in C2C12 cells **(Figure 5F)**. Next, we used Akt inhibitor (Akt i) to further examine the involvement of Akt in the treatment of Antcin K after CTX-induced muscle injury. The inhibition of IL-6, IL-1β, MuRF-1, and Atrogin-1 expression, as well as the induction of MyoD and Pax7 expression by Antcin K treatment, were reversed by Akt i in C2C12 cells **(Figure 5G─I)**. Finally, we used wortmannin and Akt i to further examine the relationship between IL-10 and PI3K/Akt signaling pathway in the treatment of Antcin K after CTX-induced muscle injury. Antcin K-produced IL-10 levels were decreased by PI3K/Akt inhibitors in CTX-induced muscle injury **(Figure 5J-K).** Therefore, after CTX-induced muscle injury, Antcin K enhances IL-10 production via the PI3K/Akt signaling pathways.

### Antcin K protects against CTX-induced skeletal muscle inflammation and injury in vivo

**Figure 6:**
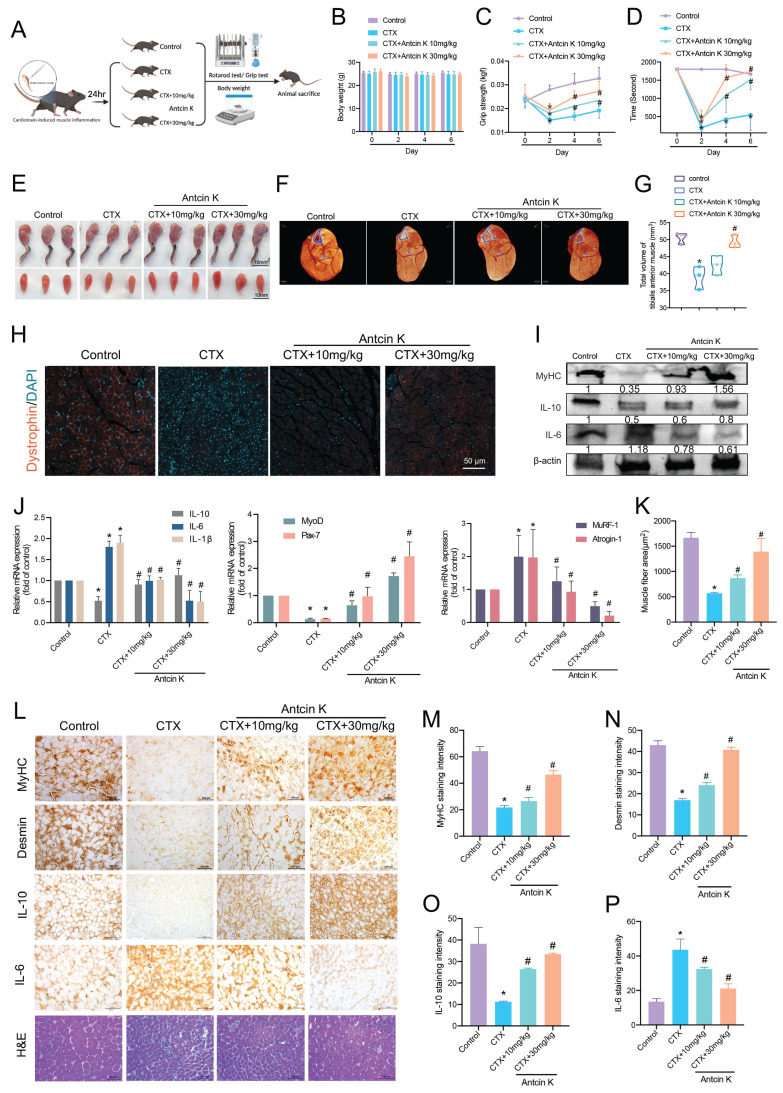
** Antcin K alleviates CTX-induced skeletal muscle inflammation and injury *in vivo*. (A)** C57BL/6 mice were randomly separated into four groups: Control, CTX, and CTX treatment with Antcin K (10mg/kg, 30mg/kg) (n=10 mice per group). **(B)** Body weight was examined on Day 0, 2, 4, and 6. **(C, D)** Functional tests involved grip strength and rotarod test.** (E)** Morphology of the legs and tibialis muscle. **(F)** After the TA muscles were stained with phosphotungstic acid for nearly two months, a micro-CT scan was conducted to analyze the TA muscle. **(G)** The total volume of TA muscle was demonstrated by micro-CT.** (H)** Dystrophin staining is represented in red coloring. Nuclei are marked by DAPI staining in blue color. **(I, J)** Western blotting and qRT-PCR were performed to analyze the expression of IL-10, IL-6, and IL-1β, as well as myogenesis markers (MyoD, Pax7) and atrophy markers (MuRF-1, Atrogin-1) in TA muscle lysates.** (K)** Quantified results from dystrophin staining. **(L)** Immunohistochemistry analysis has investigated the expression of **(M)** MyHC,** (N)** desmin, **(O)** IL-10, and **(P)** IL-6 (magnification 20×) and demonstrated TA muscle cross-sections stained with H&E. Scale bar = 100µM. *p < 0.05 compared with the control group, #p < 0.05 compared with the CTX-induced muscle injury group.

We then investigated the effects of Antcin K on short-term CTX-induced skeletal muscle inflammation and injury in a mouse model. The mice were randomly assigned to four groups: the control group, the CTX-induced muscle injury group, the CTX-induced muscle injury with low-dose Antcin K group (10 mg/kg), and the CTX-induced muscle injury with high-dose Antcin K group (30 mg/kg) (n=10 for each group)** (Figure 6A)**. TA muscles were intramuscularly injected with the CTX on Day 0, followed by another intraperitoneal injection of Antcin K low or high dose every two days. The mice were sacrificed and analyzed on Day 6. Body weight has been measured every two days, and it didn't have any significant changes between all groups **(Figure 6B)**. The results of the grip strength and rotarod test indicated a significant result in both CTX-induced muscle injury with Antcin K low and high dose groups compared to CTX group **(Figure 6C and D).** The gross hind limb musculature and TA muscles of mice were displayed for 3 mice from each group **(Figure 6E)**. Micro-CT images of the calf from hind limbs showed that the muscle thickness was significantly increased in both CTX-induced muscle injury with Antcin K low and high dose groups compared to CTX group **(Figure 6F),** and total volumes of the calf from hind limbs were quantified in **Figure 6G**. Dystrophin staining also had a similar result with micro-CT that the myofiber shapes and fluorescent intensity were clear in CTX-induced muscle injury with Antcin K high dose group compared to CTX group **(Figure 6H)**, and the fluorescent intensity was quantified based on the cross-sectional area of the TA muscles by using Image J software **(Figure 6K).** Protein expression analysis demonstrated increased MyHC and IL-10 levels and decreased IL-6 levels in the low-dose and high-dose Antcin K treatment group compared to the CTX-induced muscle injury group. **(Figure 6I).** mRNA isolated from TA muscles revealed a decrease in IL-6, IL-1β, MuRF-1, and Atrogin-1, along with an increase in IL-10, MyoD, and Pax-7 in both the low-dose and high-dose Antcin K treatment groups compared to the CTX-induced muscle injury group **(Figure 6J).** IHC staining revealed increased levels of MyHC, desmin, IL-10, MuRF-1, and CD34, along with decreased IL-6 levels, in both the low-dose and high-dose Antcin K treatment groups compared to the CTX-induced muscle injury group. **(Figure 6L and Supplementary Figure S2).** These findings suggest that myogenesis and muscle stem cell activation play a role in the muscle repair process following Antcin K treatment. The significant results from IHC were quantified **(Figure 6M─P).** In addition, we also investigated the inflammatory cell infiltration by the IHC staining to macrophages and leukocytes. The expression of CD68 and F4/80, markers of macrophages and leukocytes, was significantly reduced in both the low-dose and high-dose Antcin K treatment groups compared to the CTX-induced muscle injury group **(Supplementary Figure S3).** Our *in vivo* results demonstrated that Antcin K ameliorates CTX-induced skeletal muscle injury, inflammation and IL-10 production.

## Discussion

Sarcopenia is a progressive decline in skeletal muscle mass caused by pathological muscle atrophy, which also has a substantial effect on muscle wasting and injury 34, 35. Muscle injury-related atrophy develops in adult skeletal muscle due to various factors, including aging, cancer cachexia, and chronic metabolic diseases 36-38. Our previous study on muscle biology demonstrated that the melatonin/Pax7 axis is a crucial therapeutic target for muscle differentiation 29. In this study, we identified Antcin K, a Chinese herbal component, as a potential treatment for muscle atrophy, demonstrating effective results at a lower dose while maintaining better cell viability **(Supplementary Figure S4)**.

A previous *in vivo* study demonstrated that CTX-induced skeletal muscle atrophy and inflammation occurred within the first 3 to 5 days, followed by the onset of muscle regeneration after 14 days 39. Recent studies have reported similar findings regarding CTX-induced muscle atrophy and inflammatory infiltration occurring within 3 to 5 days; however, their treatments enhanced skeletal muscle regeneration over a 14-day period 40, 41. Our *in vivo* results demonstrated that CTX-induced skeletal muscle atrophy occurred within 5 days, with muscle regeneration not beginning until after 2 weeks. This delay in the onset of muscle regeneration may be considered a limitation of our study.

Following 5 days of muscle atrophy induction, Antcin K was administered via intraperitoneal injection every two days over a 6-day period. Our evidence indicates that Antcin K possesses anti-inflammatory properties and effectively mitigates short-term CTX-induced muscle injury in both myoblasts and myocytes. In addition, a recent study demonstrated that aging affects the immune response to cytokines and myogenesis, as evidenced by CTX-induced muscle injury in aging mice, which results in the recruitment of M2 macrophages and increased IL-10 expression 42. Since the baseline staining intensity of these markers differs significantly, we were unable to establish a direct correlation in the quantified results of the IHC staining. Our *in vivo* results also indicated that CTX-induced muscle injury led to inflammatory cell infiltration, including macrophages and leukocytes **(Supplementary Figure S3).** Antcin K holds promise as a natural compound with the potential to prevent the progression of muscular injury related to the pathophysiology of sarcopenia.

IL-10 is a regulatory cytokine known for its therapeutic role in treating inflammatory conditions such as asthma and in modulating vascular smooth muscle cells 43-45. Previous research on myoblast differentiation has shown that IL-10 plays a crucial role in regulating the shift of macrophage phenotypes, a process essential for muscle development 46. A previous histological study reported that muscle fibers from IL-10 knockout mice exhibit significantly slower recovery rates compared to those from normal wild-type mice, suggesting that the absence of IL-10 impairs muscle growth and differentiation 47. A recent study showed that IL-10 knockout mice had a significant decrease in skeletal muscle and body weight at 28 weeks 48. We also established stable IL-10 knockout C2C12 (IL-10⁻/⁻ C2C12) cell lines to validate our findings. After 7 days of incubation in DM, however, myogenic differentiation did not occur in these IL-10⁻/⁻ C2C12 clones (data not shown), suggesting that IL-10 knockout induces endoplasmic reticulum stress and contributes to skeletal muscle frailty in aged mice 49, 50. Our results emphasized the importance of IL-10 in preventing muscle wasting by counteracting the pro-inflammatory effects of IL-6, with the absence of IL-10 leading to increased IL-6 levels, muscular injury, and inflammation. However, the detailed counteracting mechanism between IL-10 and IL-6 require to be further examined in future studies. In addition, accumulating evidence suggest that cysteinyl cathepsin family plays critical role in skeletal muscle atrophy 39, 51. Specifically, we demonstrated that Antcin K mitigated cathepsin V-induced muscle atrophy by reducing pro-inflammatory markers (IL-6 and IL-1β) and atrophic markers (MuRF-1 and Atrogin-1), while enhancing myogenic markers (MyoD and Pax-7) **(Supplementary Figure S5).** These results, combined with previous studies showing the distinct roles of cathepsin S in skeletal muscle atrophy and cathepsin K in smooth muscle apoptosis 52, 53, suggest that targeting cathepsin may be a promising strategy for muscle preservation and regeneration. Thus, our study is the first to demonstrate the significant effects of Antcin K on IL-10 production, particularly in regulating anti-inflammatory processes and promoting myogenesis, implicating that targeting IL-10 might offer a potential promising therapeutic approach for treating muscle injury and sarcopenia.

Previous research has demonstrated that Antcin K effectively controlled inflammation in the RASF cells 26, and inhibited tumor metastasis in cancer through the PI3K/Akt signal pathway 25. These pathways also play essential roles in regulating IL-10 for myogenesis. For instance, Akt was involved in the IL-10 and MyoD expression in CTX-induced muscle atrophy 41. Our results showed that Antcin K enhanced the expression of IL-10 by activating the PI3K/Akt signaling pathway, suggesting its potential role in stimulating muscle synthesis through MyoD activation. In addition, accumulating evidence indicates that MyoD functions as a transcription gene that regulates muscle differentiation 54, 55. A previous sports medicine study indicated that increased levels of MyoD and myogenin, along with decreased IL-10 expression, were associated with myogenesis and muscle differentiation of myogenic precursor cells following icing treatment for acute skeletal muscle damage 56. Our results demonstrated that Antcin K enhances the expression of myogenesis markers, MyoD and Pax7, while reducing atrophy markers, MuRF-1 and Atrogin-1, through IL-10 production in CTX-induced muscle injury. These findings suggest a potential link between myogenesis and anti-inflammatory responses in the muscle repair process following Antcin K treatment. Antcin K shows promise as a therapeutic medication by regulating the expression of IL-10 through the PI3K/Akt signaling pathway and promoting myogenesis.

*A. cinnamomea* had already been documented as a widely recognized remedy in traditional Chinese medicine for cancer prevention 23. Moreover, the efficacy of Antcin K in treating type 2 diabetes and hyperlipidemia has been demonstrated in C2C12 cell lines 57, Our research emphasizes that Antcin K has substantial effects on the prevention of muscle injury associated with inflammation. The potential of Antcin K as a therapeutic strategy against sarcopenia is indicated by its capacity to inhibit inflammation and promote myogenesis. Antcin K has previously been shown for its ability to prevent rheumatoid arthritis by blocking the pro-inflammatory 24, 26. Our investigation demonstrated that Antcin K reduces inflammatory responses and protects muscle by increasing IL-10 expression through the PI3K/Akt pathway.

**Figure 7:**
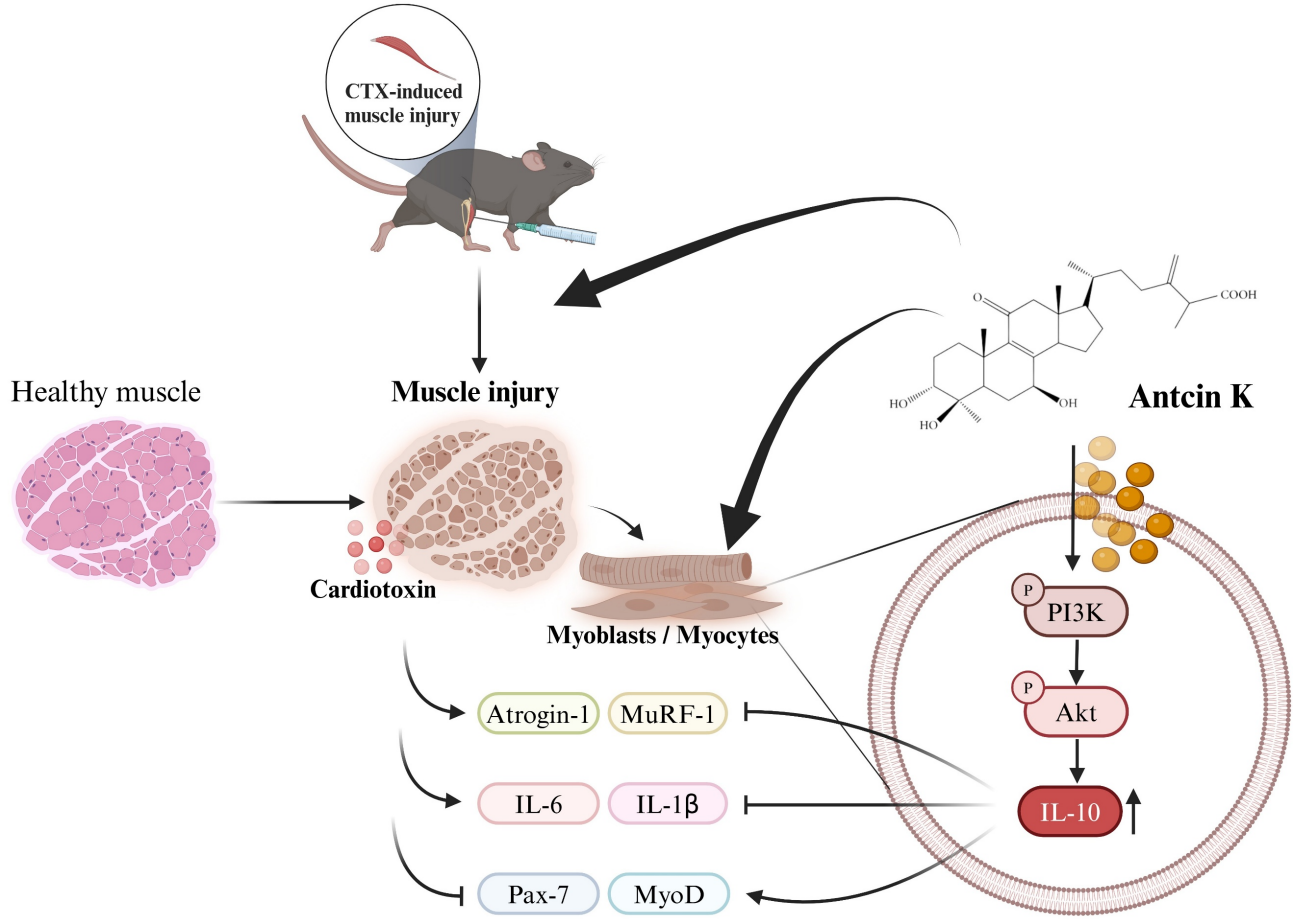
Schematic diagram displaying the function of Antcin K under CTX-induced muscle injury *in vitro* and *in vivo*.

In conclusion, our study indicates that Antcin K induces IL-10 regulation, which provides a protective anti-inflammatory effect and promotes myogenesis in the context of CTX-induced muscle injury. Antcin K improves CTX-induced muscle injury by mitigating inflammation and injury through the upregulation of IL-10 expression via the PI3K/Akt pathways in myoblasts and myocytes **(Figure 7)**. Our findings suggest that novel therapies for muscular injury, particularly through Antcin K, warrant further clinical investigations to validate its efficacy and safety in humans, with an emphasis on understanding the molecular mechanisms involved and exploring IL-10 targeting as a potential innovative approach to controlling sarcopenia and enhancing muscle mass and function in the aging population.

## Supplementary Material

Supplementary methods, figures and tables.
