# Supplementary material for: Antcin K ameliorates cardiotoxin-induced skeletal muscle injury and inflammation via IL-10 regulation
Source: Int J Biol Sci. 2025 Mar 19;21(6):2493–507. doi: 10.7150/ijbs.107343 (PMC12035881; doi:10.7150/ijbs.107343)
Supplement: Supplementary file 1 — Supplementary methods, figures and tables. [file ijbsv21p2493s1.pdf]

# Antcin K ameliorates cardiotoxin-induced skeletal muscle injury and inflammation via IL-10 regulation

Ting-Kuo Chang, Lin-Chu Huang, Yueh-Hsiung Kuo, Chun-Hao Tsai, Hsien-Te Chen, Yi-Syuan Wu, Chih-Hsin Tang*, Chen-Ming Su*

## Supplementary Materials and Methods

**Quantitative polymerase chain reaction (qRT-PCR)**

Total RNA was extracted using the TRIzol™ reagent, and 1 μg of total RNA was reverse transcribed into cDNA. qRT-PCR was performed in a 20 µL reaction volume containing cDNA template, various primers, PCR Supermix and nuclease-free water. The reaction was carried out in a StepOnePlus™ Real-Time PCR System with the following cycling conditions: 95°C for 10 minutes (polymerase activation), followed by 40 cycles of 95°C for 15 seconds and 60°C for 1 minute (fluorescence detection). All results were analyzed with StepOne software (Version 2.3). GAPDH or beta-actin was used as the internal control. All results are calculated by StepOne software version 2.3, and obtained from 6 independent experiments at least performed in duplicate.

**Western blot analysis**

All samples were lysed using RIPA buffer containing protease and phosphatase inhibitors to extract total protein, and equal amounts of protein were prepared for subsequent Western blot analysis. Briefly, the equal amounts of protein were separated by SDS-PAGE electrophoresis and then transferred to Immobilon® poly-vinylidene fluoride membrane (PVDF) membranes. Primary and secondary antibodies were applied as in Supplementary Table S2. The intensity of the blotting signals was captured and quantified using the ImageQuant™ LAS 4000 biomolecular imager (GE Healthcare, Little Chalfont, UK).

**Immunofluorescence staining**

The C2C12/Control or C2C12/IL-10-/+ mouse myoblasts or the differentiated myocytes were fixed, permeabilized, and labeled with various primary antibodies and secondary antibodies Alexa-Fluor® 488 or 594 conjugate (Thermo Fisher Scientific, UK). 4,6-Diamidino-2-phenylindole (DAPI) was used for staining nuclei. All fluorescent intensities of the indicated proteins were calculated and analyzed by using ImageJ software.

**Immunohistochemical staining**

For immunohistochemical staining, TA muscles from hind limbs of the mouse model were prepared for paraffin-embedded sections, according to previous reports [2, 3]. After the specimens were rehydrated and stained with indicated antibodies, immunohistochemistry (IHC) was performed using an IHC Kit (Sigma-Aldrich, St. Louis, MO, USA), according to the manufacturer's instructions.

**Cell viability assay**

3-(4,5-dimethylthiazol-2-yl)-2,5-diphenyltetrazolium bromide (MTT) assay was performed to assess cell viability in C2C12 and G7 cell lines following Antcin K treatment in a dose-dependent manner for 24 hours. After treatment, MTT solution (0.5 mg/mL) was added to the cells and incubated to allow formazan formation. The absorbance of formazan crystals, reflecting the number of viable cells, was measured at 550 nm using a microplate reader (Bio-Tek, Winooski, VT, USA).

# Supplementary Figures

## Figure S1

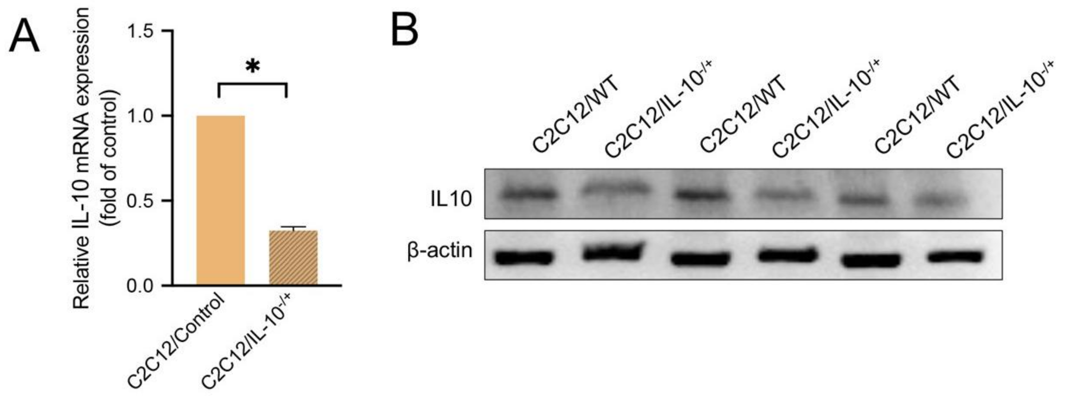


**Figure S1. The knockdown efficacy of knockdown IL-10 (IL-10$^{-/+}$) examined by qRT-PCR (A) and western blotting (B).**

## Figure S2

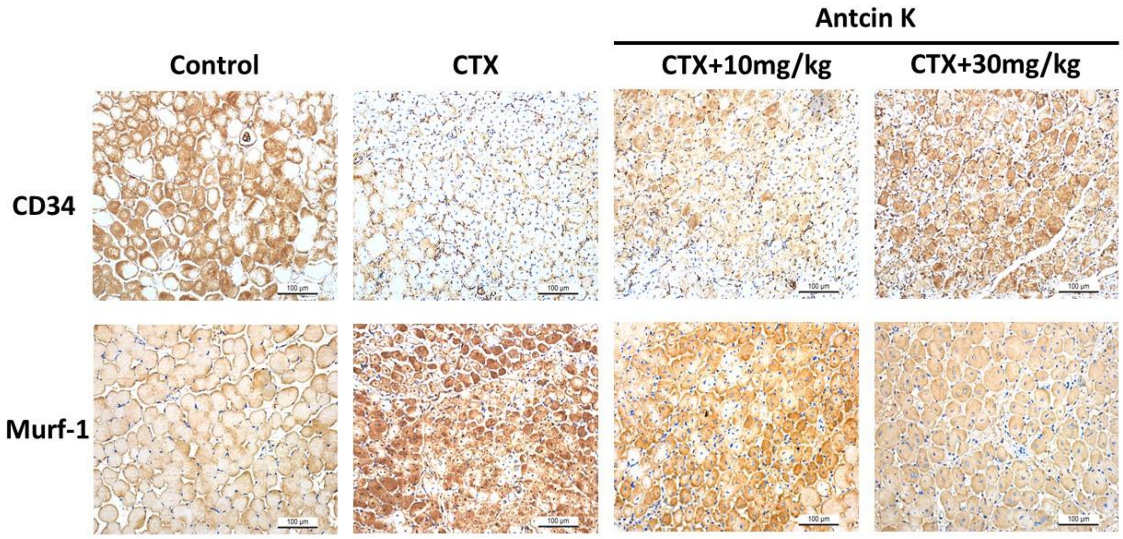


**Figure S2. IHC staining for tibialis anterior skeletal muscle *in vivo*.** CD34 is a marker of muscle stem cells. MuRF-1 is a marker of muscle atrophy.

## Figure S3

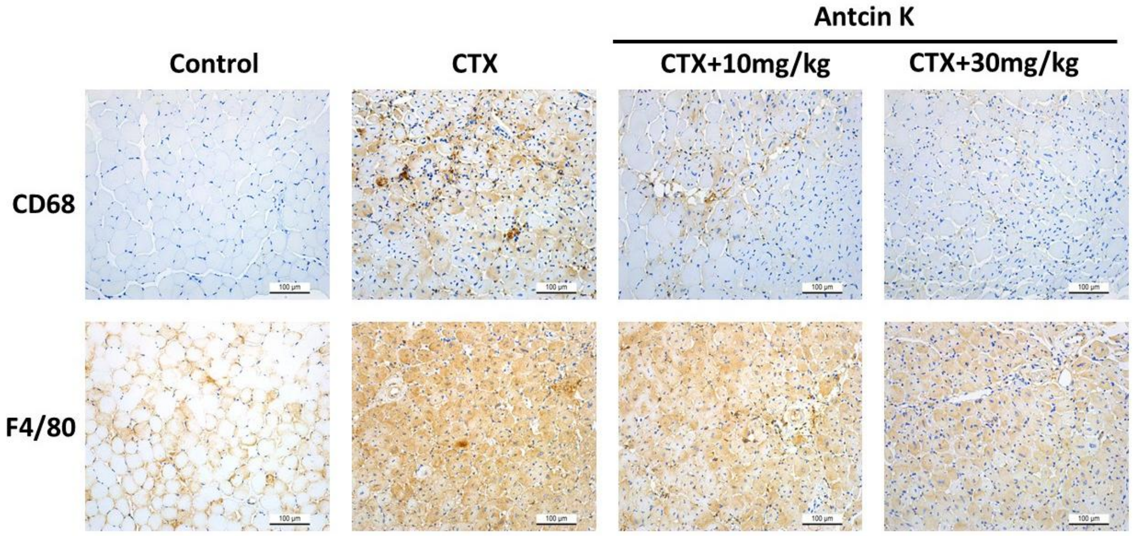


**Figure S3. IHC staining for tibialis anterior skeletal muscle *in vivo*.** CD68 is a marker of macrophages. F4/80 is a marker of leukocytes.

# Figure S4

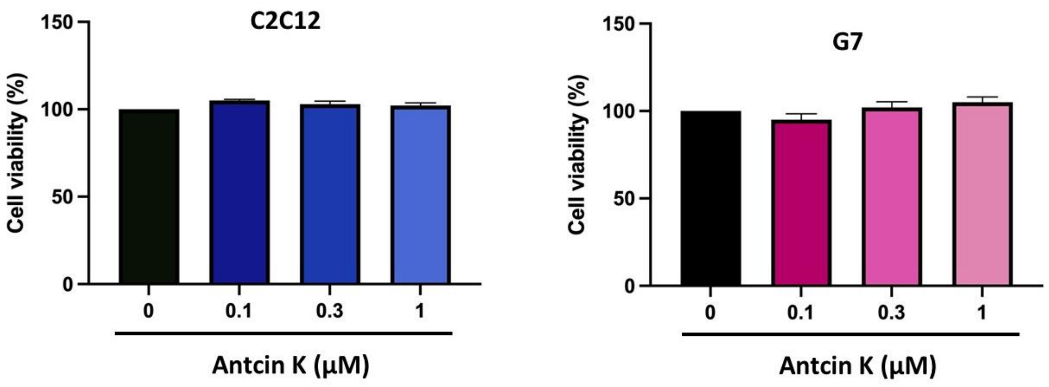


**Figure S4. MTT assay was used for the cell viability of Antcin K in C2C12 and G7 cell lines for 24 hours.**

# Figure S5

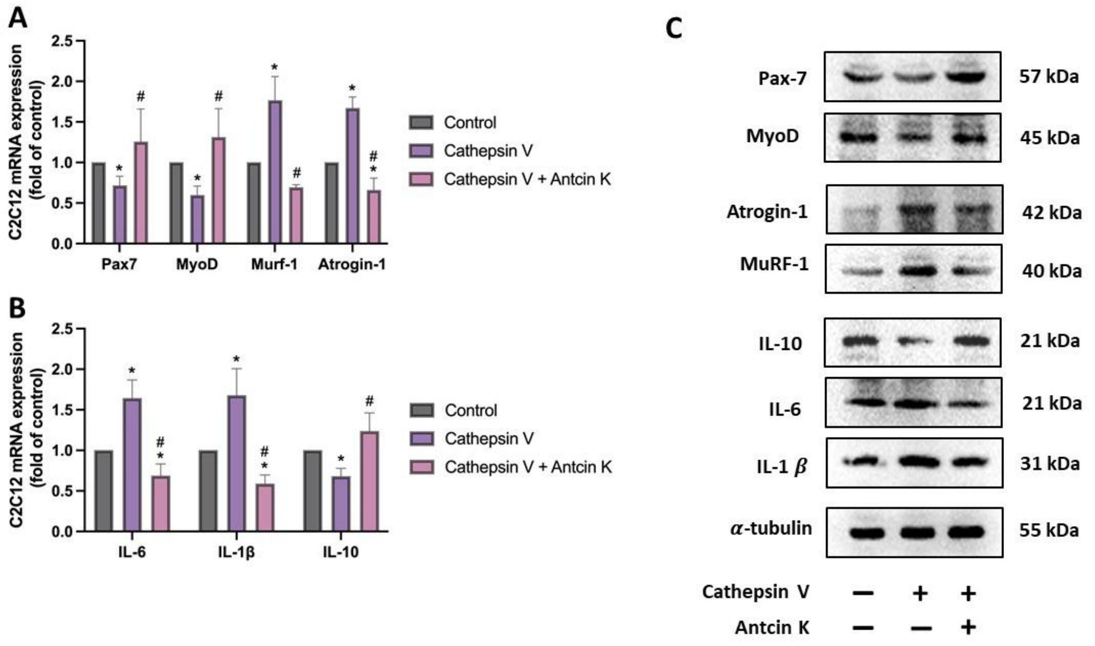


**Figure S5. Antcin K alleviated cathepsin V-induced inflammation and enhanced differentiation. (A and B)** The C2C12 was treated with the cathepsin V (10 µM) for 24 h, followed by Antcin K (1 µM) for another 24h, and the mRNA levels of proinflammatory cytokines (IL-6, IL-1β), myogenesis (MyoD, Pax-7), and atrophy markers (MuRF-1, Atrogin-1) were measured by qRT-PCR. **(C)** The protein expression of IL-10, proinflammatory markers (IL-6 and IL-1β) myogenesis markers (MyoD, Pax-7) atrophy markers (MuRF-1, Atrogin-1) was analyzed by western blotting in C2C12 cell lines.

# Supplementary Tables

**Table S1.** List of primers used in qRT-PCR

| Gene | Sequences (5'-3') |
|---|---|
| *Atrogin-1* | Forward: GAGTGGCATCGCCCAAAAGA<br>Reverse: TCTGGAGAAGTTCCCGTATAAGT |
| *MuRF-1* | Forward: GTGTGAGGTGCCTACTTGCTC<br>Reverse: GCTCAGTCTTCTGTCCTTGGA |
| *MyoD* | Forward: GAGGATCCGATGGAGCTTCTATCG<br>Reverse: CGGATCCTCTCAAAGCACCTGATA |
| *Pax-7* | Forward: GGTCC CCAGG ATGAT GAGA<br>Reverse: TTGAT GAAGA CCCCA CCAAG |
| *IL-6* | Forward: GATGGTCTTGGTCCTTAGCC<br>Reverse: GGGAAATCGTGGAAATGAGA |
| *IL-1β* | Forward: GCAACTGTTCCTGAACTCAACT<br>Reverse: ATCTTTTGGGGTCCGTCAACT |
| *IL-10* | Forward: GCTCTTACTGACTGGCATGAG<br>Reverse: CGCAGCTCTAGGAGCATGTG |
| *GAPDH* | Forward: TGTGTCCGTCGTGGATCTGA<br>Reverse: TTGCTGTTGAAGTCGCAGGAG |

**Table S2.** List of reagents, resources, and antibodies

| ANTIBODIES | HOST/ISOTYPE | SOURCE | IDENTIFIER |
|---|---|---|---|
| MyoD (G-1) monoclonal antibody | Mouse / IgG | Santa Cruz Biotech | Cat#sc-1637 |
| Pax-7 polyclonal cntibody | Rabbit / IgG | Abcam | Cat#ab187339 |
| MAFbx/Atrogin-1 (F-9) monoclonal antibody | Mouse / IgG | Santa Cruz Biotech | Cat#sc-166806 |
| MuRF-1 monoclonal antibody (C-11) | Mouse / IgG | Santa Cruz Biotech | Cat#sc-398608 |
| IL-6 polyclonal antibody | Rabbit / IgG | Genetex | Cat#GTX110527 |
| IL-1β monoclonal antibody | Rabbit / IgG | Genetex | Cat#GTX636887 |

| | | | |
|---|---|---|---|
| β-Actin monoclonal antibody | Mouse / IgG | Sigma-Aldrich | Cat#A5441 |
| Neutralizing IL-10 monoclonal antibody | Rat / IgG | R&D system | Cat#MAB417 |
| α-Tubulin monoclonal antibody | Mouse / IgG | Abcam | Cat#ab7291 |
| Myosin skeletal muscle polyclonal antibody | Mouse / IgG | R&D system | Cat#MAB4470 |
| Phospho-PI3 kinase p85(Tyr458)/p55(Tyr199) polyclonal antibody | Rabbit / IgG | Cell Signaling Tech | Cat#4228S |
| PI3-kinase p85α (B-9) monoclonal antibody | Mouse / IgG | Santa Cruz Biotech | Cat#sc-1637 |
| p-Akt1/2/3 (Thr308) monoclonal antibody | Rabbit / IgG | Santa Cruz Biotech | Cat#sc-16646-R |
| Akt1 (B-1) monoclonal antibody | Mouse / IgG | Santa Cruz Biotech | Cat#sc-5298 |
| Desmin polyclonal antibody | Rabbit / IgG | Abcam | Cat#ab216616 |
| Dystrophin polyclonal antibody | Rabbit / IgG | Abcam | Cat#ab15277 |
| Rabbit anti-goat IgG-HRP conjugated secondary antibody | Rabbit / IgG | Sigma-Aldrich | Cat#A8919 |
| Mouse anti-rabbit IgG-HRP conjugated secondary antibody | Mouse / IgG | Santa Cruz Biotech | Cat#sc-2357 |
| Goat anti-rabbit IgG (H+L), Alexa Fluor 488 conjugated secondary antibody | Goat / IgG | Thermo Fisher Scientific | Cat#A32731 |
| Goat anti-rabbit IgG (H+L), Alexa Flour 594 conjugated secondary antibody | Goat / IgG | Thermo Fisher Scientific | Cat#A32740 |
| CD34 | Rabbit / IgG | Abcam | Cat#ab81289 |
| CD68 | Mouse / IgG1 kappa | Novus Biologicals | Cat#NB100-683 |
| F4/80 | Mouse / $IgG_1$ | Santa Cruz Biotech | Cat#SC-377009 |

| CHEMICALS, PLASMID | SOURCE | IDENTIFIER |
|---|---|---|
| wortmannin (PI3K inhibitor) | Santa Cruz Biotech | Cat#SC-3505A |
| Akt inhibitor | Sigma-Aldrich | Cat#A6730 |
| Mouse shRNA IL-10 plasmid | National RNAi Core Facility (Sinica, Taiwan) | Cat#TRCN0000365913 |
| CMV promoter plasmid | Addgene | Cat#169739 |
| MD plasmid | Addgene | Cat#20864 |
| DAPI | Sigma-Aldrich | Cat#D9564 |
| Cardiotoxin | LATOXAN | Cat#L8102-1MG |
| Immobilon Western Chemiluminescent HRP substrate | Merck millipore | Cat#WBKLS0500 |
| IHC Kit | Sigma-Aldrich | Cat#00-4955-58 |
| Cathepsin V overexpression | National RNAi Core Facility (Sinica, Taiwan) | Cat#NJ0090248-1 |
| **CELL LINES** | **SOURCE** | **IDENTIFIER** |
| C2C12 mouse myoblast | ATCC | Cat#CRL-1772 |
| G7 mouse myoblast | ATCC | Cat#CRL-1447 |
| 293 T | Thermo Fisher Scientific | Cat#R700007 |
| **CULTURE MEDIUM** | **SOURCE** | **IDENTIFIER** |
| Dulbecco’s Modified Eagle’s Medium (DMEM) | Gibco | Cat#41966-029 |
| Fetal bovine serum (FBS) | Gibco | Cat#21875-034 |
| Horse serum (HS) | Gibco | Cat#16050-122 |
| Trypsin | Gibco | Cat#15090-046 |
